# Two Test Assembly Methods With Two Statistical Targets

**DOI:** 10.3389/fpsyg.2022.786772

**Published:** 2022-02-11

**Authors:** Zheng Huijing, Li Junjie, Zeng Pingfei, Kang Chunhua

**Affiliations:** Key Laboratory of Intelligent Education Technology and Application of Zhejiang Province, Zhejiang Normal University, Jinhua, China

**Keywords:** bimodal distribution, item matching test assembly methods, item response theory, information curve, parallel forms of tests

## Abstract

In educational measurement, exploring the method of generating multiple high-quality parallel tests has become a research hotspot. One purpose of this research is to construct parallel forms item by item according to a seed test, using two proposed item selection heuristic methods [minimum parameters–information–distance method (MPID) and minimum information–parameters–distance method (MIPD)]. Moreover, previous research addressing test assembly issues has been limited mainly to situations in which the information curve of the item pool or seed test has a normal or skewed distribution. However, in practice, the distributions of information curves for tests are diverse. These include multimodal distributions, the most common type of which is the bimodal distribution. Therefore, another main aim of this article is to extend the information curves of unimodal distributions to bimodal distributions. Thus, this study adopts simulation research to compare the results of two item, response, theory (IRT)-based item matching methods (MPID and MIPD) using different information curve distributions for item pools or seed tests. The results show that the MPID and MIPD methods yield rather good performance in terms of both two statistical targets when the information curve has a unimodal distribution, and two new methods yield better performance than two existing methods in terms of test information functions target when the information curve has a bimodal distribution.

## Introduction

Constructing multiple equivalent forms with higher quality to be administered at different timepoints and locations has always posed a challenge for developers of educational assessments and licensure tests. The application of automated test assembly (ATA) procedures benefits test developers in that it dramatically reduces their workload and ensures the quality of parallel test forms. Over the past two decades, researchers have successfully implemented optimization-based automated test assembly techniques such as mixed integer programming (MIP; Cor et al., 2009; Finkelman et al., 2010) and enumerative heuristics (Armstrong et al., 1992; Finkelman et al., 2009; Brusco et al., 2013).

The MIP methods convert test specifications (the test blueprint) into mathematical expressions from which a globally optimal solution can usually be derived using available software packages (Chen, 2014). Heuristics methods following stepwise procedures are of great influence even though they often yield a locally optimal solution at each step, not a globally optimal one (Chen, 2014). Because of the nondeterministic polynomial (NP)-hard nature of MIP problems, heuristic methods can improve both the performance of MIP solvers and the quality of solutions (Chen, 2015).

Mixed integer programming approaches look for the optimal solution, so time is longer. Besides, many solvers are commercially available and costly. For users with a weak mathematical background, MIP approaches are not easily accessible (Chen, 2014). Heuristic methods avoid the above shortcomings. Although heuristic methods find the suboptimal solution, the suboptimal solution is acceptable for test assembly, so this article focuses on heuristic methods. There are many heuristics (Armstrong et al., 1992; Finkelman et al., 2009; Brusco et al., 2013), but most of them like greedy algorithms, random and sampling algorithms are relatively old algorithms, which are difficult to meet today’s demand for test papers with diverse constraints. With the development of test theory, the trend of test assembly is to assemble high-quality test papers that meet the constraints under the test theory framework based on seed test. Minimum information distance method (MID) and minimum parameters distance method (MPD) are two classical test assembly methods based on seed test under item response theory.

When the seed test is available, one of the targets of test assembly is to make test information curve of generated tests similar to test information curve of the seed test, because an important indicator for testing whether two tests are parallel tests, is the similarity of test information curves of the two tests (Ali and Van Rijn, 2016). The more similar they are, the more they can be regarded as parallel tests. The core idea of the MID method is to match item information curve item by item, so that the test information curve of the seed test and generated tests will be identical (Armstrong et al., 1992). Another indicator is the test characteristic curve of two tests (Ali and Van Rijn, 2016). Similar test information does not necessarily guarantee that the test characteristic curve is the same. So the advantage of MID is that the generated tests are similar to the seed test in terms of test information curve, but the disadvantage is that the test characteristic curve is not necessarily similar.

In order to meet both the two indicators, Armstrong et al. (1992) have attempted to use the MPD method for directly matching the item’s parameters, because test information function and test characteristic function are both functions composed of some parameters, which will inevitably be decided by parameters.

In general, MID only focuses on test information curve, while MPD has a wide range of influence. It can be inferred that MID is better than MPD on test information curve matching target, while MPD is better than MID on test characteristic curve matching target (Armstrong et al., 1992). On the basis of MID and MID, can new test assembly methods be produced to make both test information curve and test characteristic curve matching targets achieve more satisfactorily?

Moreover, the majority of previous research addressing test assembly problems has focused on the condition when the information curve of the item pool or the reference test has a unimodal distribution by default (Chen et al., 2012; Chen, 2014, 2015; Ali and Van Rijn, 2016; Shao et al., 2019). However, information curves vary greatly in practice, and they include both unimodal and multimodal distributions. The bimodal distribution is a simple and typical representative of the multimodal distribution. Accordingly, the present study explores both unimodal and bimodal distributions. In sum, this study’s goal is to develop two novel item, response, theory (IRT)-based item matching test assembly methods based on the two previously mentioned methods and then compare the four, using different information curve distributions for the item pools and seed tests.

The article is organized as follows. First, we briefly review two extant item matching test assembly methods (the minimum parameters–distance method and the minimum information–distance method), explaining their limitations and proposing two new methods. Subsequently, we introduce information curves for unimodal and bimodal distributions. Finally, we compare the proposed methods with the two traditional item matching methods, using different information curve distributions for the item pools and seed tests based on several criteria.

## Two Traditional Heuristic Methods

### Minimum Information Distance Method

**FIGURE 1:**
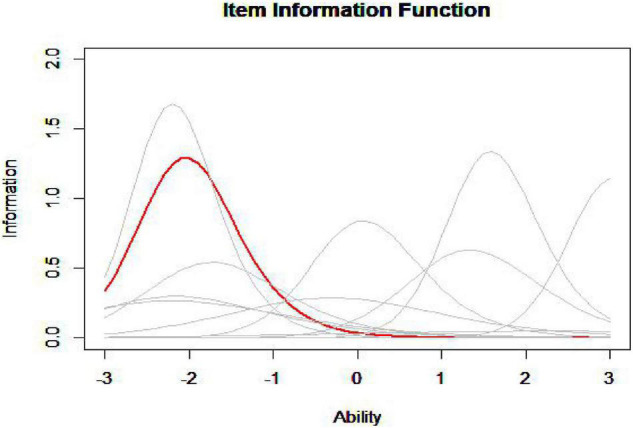
Item information function.

The idea of the MID method is to find one item in the item pool that is most similar with the item in the seed test in terms of item information curve. The figure given below (Figure 1) is the information curve of all the items in the item pool (gray curve) and an item in the seed test (red curve).

According to the image, it is hard to judge which item in the item pool has the closest information curve to the item in the seed test, so it is necessary to calculate the information curve distance (ID) between each item in the item pool and the item in the seed test to find the minimum ID (MID), and this is in line with the original intention of the MID method to assemble tests.


$$I\,D_{i\,j}^{2}=\sum_{m=1}^{M}\omega_{m}\,{\left(f_{i}\,\left(\theta_{m}\right)-f_{j}\,\left(\theta_{m}\right)\right)}^{2}$$

The information distance is estimated by the following equation:

where *ID*_*ij*_ is the information distance between item *i* (in the seed test) and item *j* (in the item pool). *f*_*i*_(θ) is the information for item *i* at the ability level θ and *f*_*j*_(θ) is the information for item *j* at the ability level θ. *M* is the number of the ability levels of interest,ω_*m*_ is weight coefficient and $\sum_{m=1}^{M}\omega_{m}=1$, ω_m_ > 0, ω_m_ is selected by the practitioner (Armstrong et al., 1992). The ability level θ selected in this study are –2, –1, 0, 1, 2, and the weight of each ability point is 0.2, the same for both.

The IRT model used in this study is a three-parameter logistic model, so the calculation formula for item information curve *f*(θ) is as follows:


$$f\,\left(\theta \right)=\frac{1.7^{2}*a^{2}*\left(1-c\right)}{\left(c+e^{1.7\,a\,\left(\theta -b\right)}\right)\,{\left(1+e^{-1.7\,a\,\left(\theta -b\right)}\right)}^{2}}$$

where *a, b* and *c* are discrimination, difficulty, and guessing parameters of an item, respectively.

The core objective of the MID method is to minimize the differences in information statistics at crucial ability points between the assembled test forms and the seed test, which directly meets the criterion of test information matching.

### Minimum Parameters Distance Method

In addition to matching the test information curve (TIC) of the seed test, matching the test characteristic curve (TCC) of the seed test is another important target of test assembly (Ali and Van Rijn, 2016). Constraining the test information curve to be equal does not necessarily guarantee similarity of the test characteristic curve (Ali and Van Rijn, 2016). It can be concluded that the MID method can only meet the matching requirements of TIC but cannot meet the matching requirements of TCC.

The IRT model used in this study is a three-parameter logistic model, so the calculation formula for item characteristic function is as follows:


$$P\,\left(\theta \right)=c+\frac{1-c}{1+e^{\left[-1.7\,a\,\left(\theta -b\right)\right]}}$$

where *a*, *b*, and *c* are discrimination, difficulty, and guessing parameters of an item, respectively.

It can be concluded from the calculation formulas of item characteristic curve (ICC) and item information curve (IIC) that they are both functions of three parameters. The idea of the MPD method is to find one item in the item pool that is most similar with the item in the seed test in terms of item’s parameters. Tests that match based on collective indices such as test, information, function (TIF) may not be presumed to exhibit stable, similar properties any more than can those based on item matching. Tests built by matching item parameters (MIP) directly capture the main properties of the items in the seed test, thereby ensuring the satisfaction of all cumulative indices, including TIFs and TCCs (Chen, 2015).

**FIGURE 2:**
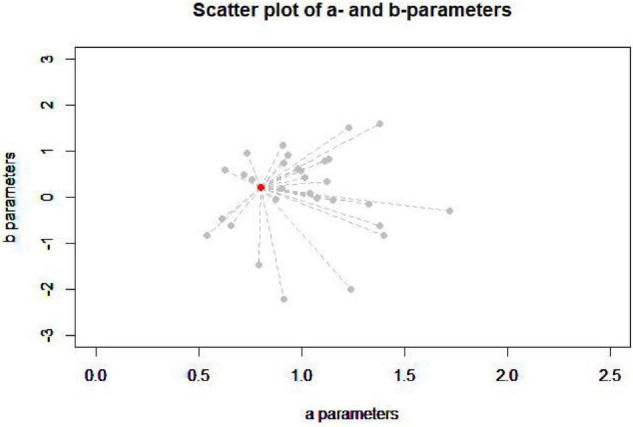
Scatter plot of *a*- and *b*-parameters.

The figure below (Figure 2) is the item’s parameters of all the items in the item pool (gray dot) and one item in the seed test (red dot).

According to the image, it is difficult to judge which item in the item pool has the closest item’s parameters to the item in the seed test, so it is necessary to calculate the item’s parameters distance (PD) between each item in the item pool and the item in the seed test to find the minimum PD (MPD), and this is in line with the original intention of the MPD method to assemble tests (Wang et al., 2016). The IRT model in this study is a commonly used three-parameters logistic model. The PD is estimated by the following equation:


$$P\,D_{i\,j}^{2}=\varphi_{1}\,{\left(a_{i}-a_{j}\right)}^{2}+\varphi_{2}\,{\left(b_{i}-b_{j}\right)}^{2}+\varphi_{3}\,{\left(c_{i}-c_{j}\right)}^{2}$$

where *PD*_*ij*_ is the parameter’s distance between item *i* and item *j*; *a*_*i*_, *b*_*i*_ and *c*_*i*_ are the discrimination, difficulty and guessing parameters, respectively, of item *i* in the seed test; and *a*_*j*,_ *b*_*j*_, and *c*_*j*_ are the discrimination, difficulty, and guessing parameters, respectively, of item *j* in the item pool.

φ_1_, φ_2_, and φ_3_ are weight coefficient. φ_1_ ≥ 0, φ_2_ ≥ 0, φ_3_ ≥ 0 and φ_1_ + φ_2_ + φ_3_ = 1. They are selected by the practitioner (Armstrong et al., 1992; Chen, 2015; Wang et al., 2021). Different parameters have different effects on the test information function and test characteristic function. Taking the three-parameters logistic model as an example, for test information function, the degree of discrimination and guessing parameters have a greater impact on it, while for test characteristic function, the degree of discrimination has the greatest influence, followed by the difficulty and guessing parameters. Therefore, when calculating the parameter distance, different weights are generally given to the parameters. Chen (2017) found that these weights (φ_1_ = 0.5, φ_2_ = 0.25, and φ_3_ = 0.25) were used to represent the relative importance of a parameter to the information function after examination of the TIC and TCC resulting from the unweighted and weighted versions. The weights used in this study are the same.

### Test Assembly Procedure

1. Randomly select an item in the seed test.
2. Choose five items (the number of items is determined by the number of parallel tests) from the item pool according to MID or MPD.
3. Five items are randomly assigned to five parallel tests and calculate the sum of the distances between the selected items of the five parallel tests and the seed test.
4. Delete the selected item from the item pool to prevent repeated selection.
5. Randomly select another item in the seed test again and choose five items from the new item pool according to MID or MPD.
6. The five items are allocated to five parallel tests based on the sum of distances (procedure three). The principle is that the greater the sum of the distances of parallel paper, the more priority items with a smaller MID or MPD are to be assigned to it, so as to reduce the difference between parallel tests.
7. Repeat 4–6 until all the items in the seed test have been selected.

**FIGURE 3:**
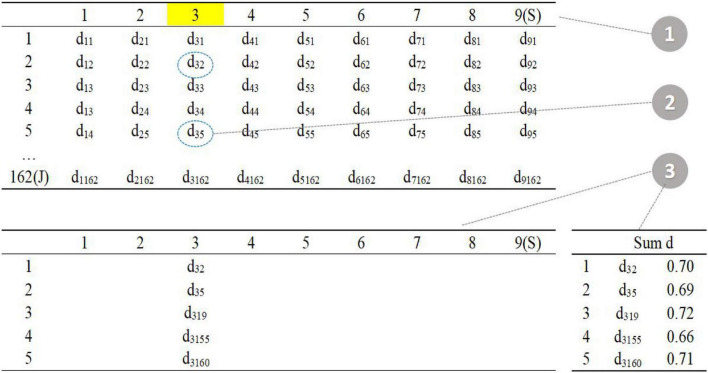
Procedure 1–3.

As shown in the figure above (Figure 3), the upper left corner is the distance matrix between the item in the item pool (row) and the item in the seed test (column); the lower left corner is the distance matrix of five parallel tests; the lower right corner calculates the sum of the current distances of each parallel test.

The first step is to randomly select one item in the seed test (item 3 in the seed test), the second step is to find the five items with the smallest *d* value in the item pool (item 2, item 5, item 19, item 155, and item 160 in the item pool), and the third step is randomly assigned to five parallel tests, and the total distance is calculated.

**FIGURE 4:**
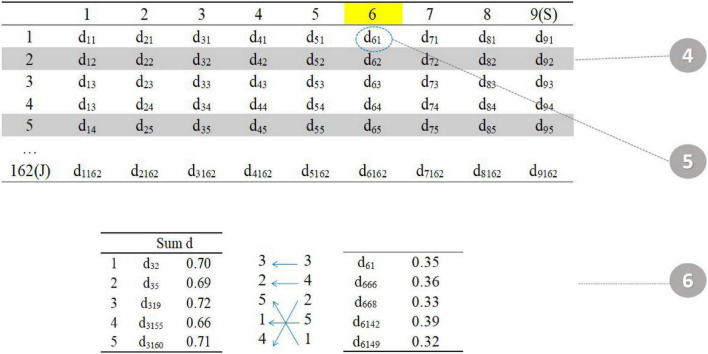
Procedure 4–5.

The fourth step is to randomly select one item in the seed test (item 6). The fifth step is to find the five items with the smallest value of d in the item pool (item 1, item 66, item 68, item 142, and item 149 in the item pool). The sixth step is to assign five items. The total distance calculated in the third step is allocated to the five parallel tests in reverse order (the smaller distance item is assigned to the test with larger total distance) to reduce the difference between parallel tests (Figure 4).

**FIGURE 5:**
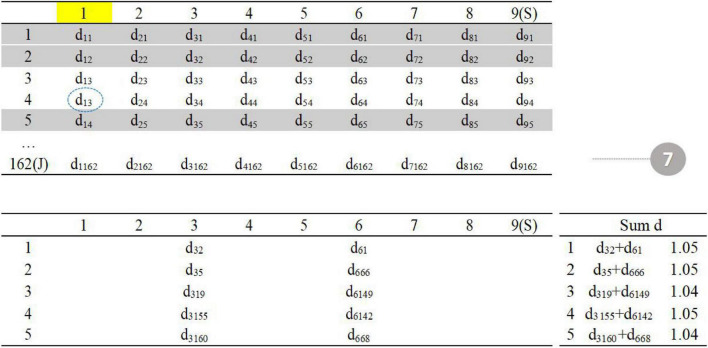
Procedure 7.

Repeat steps 3–6 until all items in the seed test have been selected (Figure 5).

### Two New Heuristic Methods

The MID method aims to make generated tests similar to the seed test in terms of the test information curve, but fails to take test characteristic curve into account. MPD method of directly matching parameters expects to achieve two targets, but the result of test information curve is inferior to method MID. The two methods have their own strengths, so why not combine the two distances together to construct a new distance index to assemble tests?

### Minimum Parameters–Information–Distance Method

In order to achieve the best result of TIF and TCC target matching, the two methods are combined when constructing the distance moment. The parameters–information–distance (PID) is estimated by the following equation:


$${P\,I\,D}_{i\,j}^{2}=\left(1-\lambda \right)*\left({P\,D}_{i\,j}\right)+\lambda *\left({I\,D}_{i\,j}\right)$$

$$\lambda =\frac{s-1}{t\,e\,s\,t\,\_\,l\,e\,n\,g\,t\,h}$$

where *PID*_*ij*_ is the combined distance between item *i* (in the seed test) and item *j* (in the item pool); *PD*_*ij*_ is the parameter’s distance between item *i* and item *j*; *ID*_*ij*_ is the information distance between item *i* and item *j*; λ is the adjustment factor, and *s* is the number of items that have been selected so far.

The progressive method of Revuelta and Ponsoda (1998) is used as a template for our holistic item selection index. The role of λ is to select and generate papers in the previous stage in order to highlight the advantages of the MPD method and match the TCC. As *s* increases, it becomes larger and 1-s decreases, highlighting the advantages of the MID method and matching the TIC at the later stage.

### Minimum Information–Parameters–Distance Method

It remains unknown whether the two methods’ sequence affects test assembly results. It is feasible to reverse the order, producing a minimum information–parameters–distance (MIPD) method for meeting a variety of practical demands. At the first stage, the purpose of selecting items is to obtain smaller TIC differences, and during the next stage, the aim is to minimize parameters’ distances. The procedure is exactly the opposite of the MPID method. The information, parameters, and distance (IPD) is estimated by the following equation:


$${I\,P\,D}_{i\,j}^{2}=\left(1-\lambda \right)*\left({I\,D}_{i\,j}\right)+\lambda *\left({P\,D}_{i\,j}\right)$$

$$\lambda =\frac{s-1}{t\,e\,s\,t\,\_\,l\,e\,n\,g\,t\,h}$$

The meaning of the letters in the formula is the same as above.

### Bimodally Distributed Test Information Curves

Bimodal distributions often appear in the fields of biology, life sciences, geology, and so on. For example, in a clinical context, the highest incidence of fibrolamellar hepatocellular carcinoma (FLC) occurs between ages 15 and 19 and between ages 70 and 74; that is, the curve representing the age of onset is bimodal (Ramai et al., 2021). Of course, bimodal distributions are not uncommon in the fields of psychology and pedagogy. Bimodal distributions appear in many psychological tests (Steinley and McDonald, 2007). This often occurs with education examinations. Tang (2018) has found that students’ English subject test scores in each semester exhibit abnormal bimodal distributions (based on the Academic Quality Monitoring and Evaluation Department).

Different disciplines have different definitions of bimodal distributions. In this article, we are referring to a distribution showing two obvious peaks—that is, a mixed distribution composed of two unimodal distributions—where the two peaks need not be equal.

**FIGURE 6:**
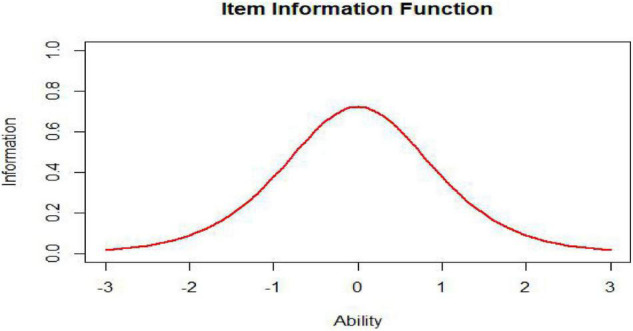
Item information function (*a* = 1, *b* = 0).

As we can see from the formula of the item information function, it is not surprising that the item information curve has one peak, such as that shown in Figure 6. An item measures the ability with greatest precision at the ability level corresponding to the item’s difficulty parameter. The amount of item information decreases as the ability level departs from the item difficulty and approaches zero at the extremes of the ability scale.

Because a test is used to estimate an examinee’s ability, we can also obtain the amount of information yielded by the test at any ability level. A test is a set of items; therefore, the test information at a given ability level is simply the sum of the item information values at that level. Consequently, the test information function is defined as


$$I\,\left(\theta \right)=\sum_{j=1}^{J}I_{j}\,\left(\theta \right)$$

where *I*(θ) is the amount of test information at ability level θ, *I*_*j*_(θ) is the amount of information for item *j* at ability level θ, and *J* is the number of items in the test.

**FIGURE 7:**
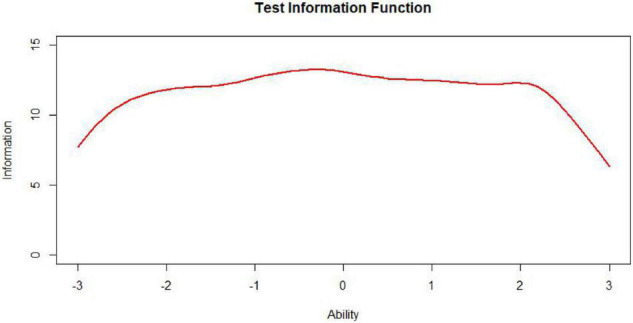
Unimodally distributed test, information, function (TIF).

**FIGURE 8:**
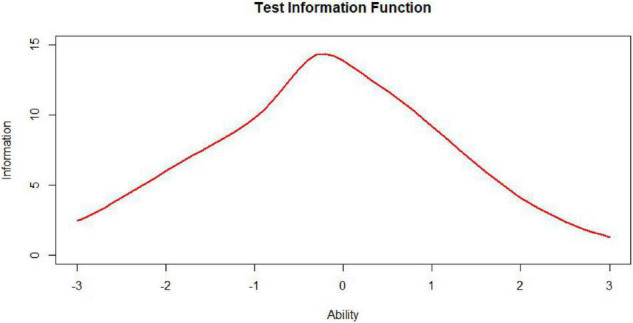
Uniformly distributed test, information, function (TIF).

**FIGURE 9:**
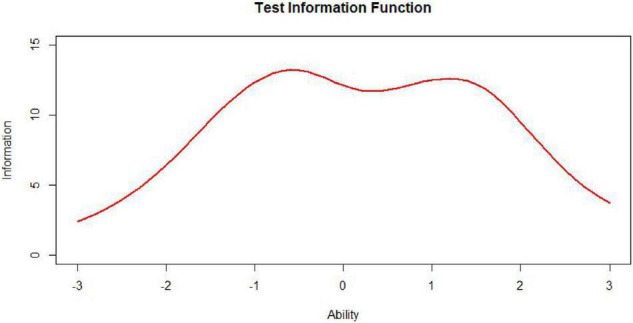
Bimodally distributed test, information, function (TIF).

The test information function is an extremely useful feature of item response theory. It provides a metric of how well the test is doing in estimating ability over the range of ability scores (Xiong et al., 2002). While the ideal test information function often may be a horizontal line (Figure 7, *n* represents test length), it may not be optimal for meeting specific demands. For example, if one aims to construct a test to award scholarships, this ideal function may not be appropriate. In this situation, one aims to measure ability with considerable precision at ability levels near that used to separate those who will receive the scholarship from those who will not. The best test information function in this case would have a peak at the cutoff score (Figure 8; Baker and Kim, 2017). Other specialized uses of tests could require different test information functions. For example, for a test provided to award scholarships at several levels, the satisfactory test information function would have multiple peaks at the cutoff scores (a multimodal distribution). The bimodal distribution is one of the simplest types (Figure 9).

Nevertheless, to our knowledge, there is little research specifically on the information curves of bimodal distributions in the context of automated test assembly. In some educational measurement, not only is it required to have a demarcation score with small error and strong discrimination at the boundary between qualified and unqualified, but also hope to have another demarcation score with small error and strong discrimination at the boundary between excellent and non-excellent. This requires that the target state of the test information function be designed as a bimodal curve (Chen and Wang, 2010). It is undoubtedly worthwhile to investigate the performance of test assembly methods based on the item pool information curves of bimodal distributions.

## Method

The goal of the simulation study was to investigate the performance of four item selection methods under various conditions:

*Pool size, test length, and number of forms:* The size of the item pool was 540, the test length was 30, and the number of parallel tests was 5.

*Item parameters:* Each item was subject to 3PLM, the discrimination parameter had a normal distribution, with a mean value of 1 and a standard deviation of 0.3; the difficulty parameter had a bimodal distribution, and the guessing parameter had a [0, 0.3] uniform distribution.

*Non-statistical constraints.* The items in the item pool covered three content areas A, B, and C, whose proportions of the total content were 40, 30, and 30%, respectively. The seed test consisted of 30 items (content proportions identical to this in item pool).

*Variables for bimodal curve of test information.* The most common bimodal distribution is a combination of two normal distributions. Xu et al. (2013) first used Excel to randomly generate two normally distributed datasets with a seed size of 1,000 and then extracted *n* × 1,000 random datapoints from the first normal distribution and 1,000 – *n* × 1,000 random datapoints from the second normal distribution (*n* is a ratio ranging from 0 to 1). They extracted random datapoints from the seed and then created a scatterplot and a histogram based on the extracted data to obtain the bimodal distribution’s shape. The procedure for producing bimodally distributed TIC is similar to the preceding process, except it is a combination of two unimodally distributed TICs. Different values of *a* will generate bimodal distributions of different shapes. In Kim and Lee’s (2020) study, the ratios of extraction from the normal component θ∼ N (–1.8, 0.8) and the normal distribution θ ∼ *N* (0.8, 0.8) were 3:7, 5:5, and 7:3, composing three different bimodal distributions. These three ratios can effectively represent the different forms of the bimodal distribution. Therefore, in this study, we set the mixing ratios of the bimodally distributed TICs to 3:7, 5:5, and 7:3. In addition to the mixing ratio, the horizontal spacing between the double peaks will also affect the shape of the bimodal curve. For this reason, we included the bimodal horizontal distances of the TICs in our estimates, which we set to 0, 1, 2, and 3. Simply put, the main variables of bimodal TICs observed in this study were the bimodal mixing ratio and bimodal horizontal spacing.

We repeated the test for *R* (1,000) times, each time randomly generating the item pool parameters and seed tests that met the preceding requirements, using the four item selection methods to generate 5 parallel test papers. To accommodate the content constraints of the test, we only directly determined the most matching items from each content sub-item pool and did not use weighting factors.

### Evaluation Criteria

1. Mean square deviation indicator of test information function (*MSD*_*TIC*_)
  We used this indicator to evaluate the difference between the assembled test and the seed test in terms of their TICs. We calculated it using the following formula:

$${M\,S\,D}_{T\,I\,C}=\left(\sum_{\text{n}=1}^{N}{\left(I_{\left(\theta_{n}\right)}-I_{\text{s}\,\left(\theta_{n}\right)}\right)}^{2}\right)/N$$

where *I*_(θ*n*)_ and *I*_*s*(θ*n*)_ represent test information of the assembled test and the seed test at ability point θ_*n*_ (*n* = 1, 2,…, *N*), respectively. The number of capability nodes *N* was set to 61, the capability range was –3 to 3, and the step size was 0.1.
2. Mean square deviation indicator of test characteristic curve (*MSD*_*TCC*_)
  This indicator was used to evaluate the difference between the TCCs of the assembled test and the seed test. We calculated it using the following formula:

$${M\,S\,D}_{T\,C\,C}=\left(\sum_{\text{n}=1}^{N}{\left(C_{\left(\theta_{n}\right)}-C_{\text{s}\,\left(\theta_{n}\right)}\right)}^{2}\right)/N$$

where *C*_(θ*n*)_ and *C*_*s*(θ*n*)_ represent TCCs of the assembled test and the seed test at ability point θ_*n*_ (*n* = 1, 2,…, N), respectively. The number of capability nodes *N* was set to 61, the capability range was –3 to 3, and the step size was 0.1.

## Results

The mixing ratio of the bimodal distribution has little effect on the results, so to avoid cluttering the presentations, the following only shows the results with a bimodal mixing ratio of 3:7. The Supplementary Appendix presents the rest of the results for interested readers. *D* represents the two peaks’ horizontal spacing.

### Test Information Curve

Table 1 lists the mean values for the mean square deviation of the five forms from the target test information at 61 ability points for all test assembly methods.

**TABLE 1:** *MSD*_TIC_.

	Unimodal (*D* = 0)	Unimodal (*D* = 1)	Bimodal (*D* = 2)	Bimodal (*D* = 3)
MPD	0.401	0.408	0.426	0.438
MID	0.302	0.331	0.363	0.406
MPID	0.296	0.315	0.339	0.348
MIPD	0.286	0.309	0.335	0.350

As Table 1 illustrates, when the TIC has a unimodal distribution, the MID method performs better than the MPD methods and the MPID and MIPD methods achieve the same *MSD* as the MID method. Furthermore, the MPD method rivals the MID methods gradually and the MPID and MIPD method perform best when the TIC has a bimodal distribution.

In sum, the MPID and MIPD method—regardless of the bimodal horizontal distance—perform the best among all the four methods. The performance of the MID method when the TIC has two peaks is not as good as when the TIC has a single peak, indicating that the MPID and MIPD method (especially the former) are much more suited for use with bimodally distributed TIC than is the MID method. The MPD method has no advantage in TIC.

van der Linden (2005) argues that if the information function curves of the two tests are very similar—that is, when the difference in the amount of information between the assembled test and the seed test at different abilities is small—then the two tests can be considered statistically equivalent. The proximity of the TCCs can also be used as an evaluation criterion for the quality of the assembled test. Plotting the test information function and test characteristic curve for the assembled test and the seed test, one can intuitively judge the pros and cons of the item selection methods (Wang et al., 2021). Due to limited space, we show only some of the results here.

**FIGURE 10:**
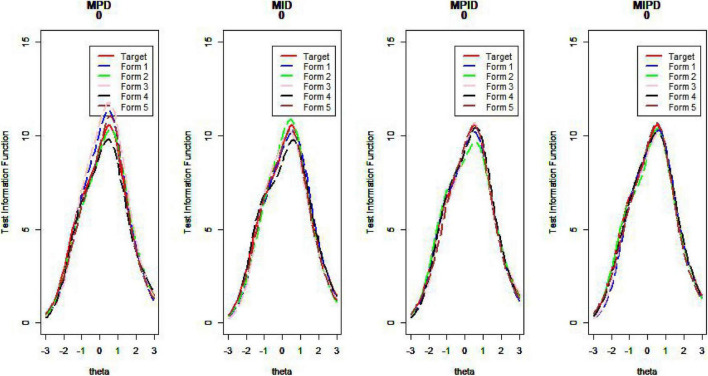
Test information curve (*D* = 0).

**FIGURE 11:**
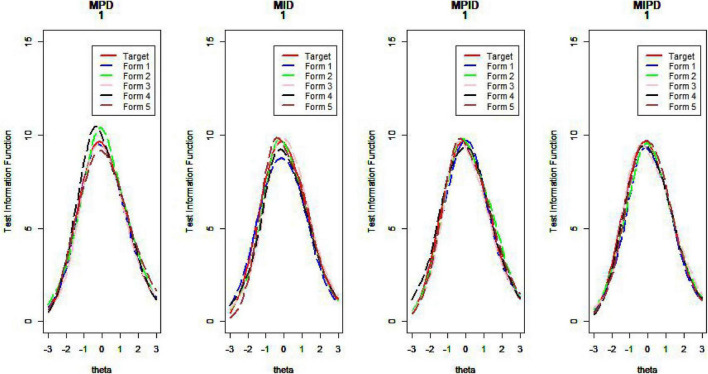
Test information curve (*D* = 1).

**FIGURE 12:**
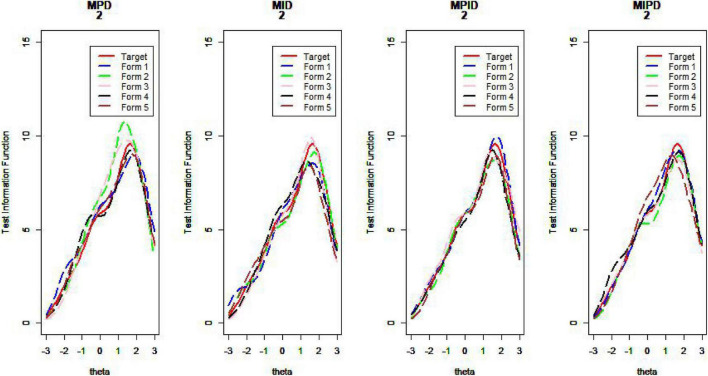
Test information curve (*D* = 2).

**FIGURE 13:**
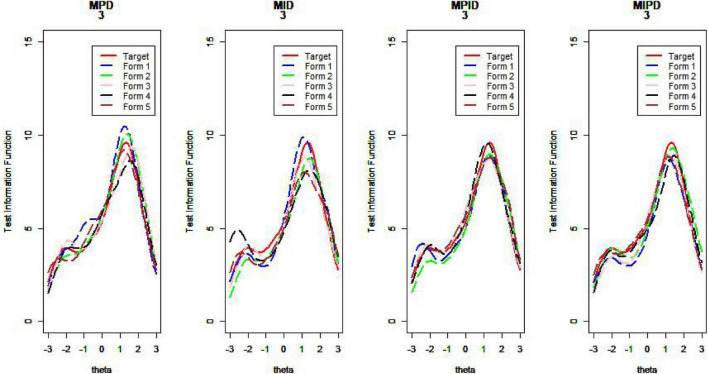
Test information curve (*D* = 3).

Figures 10–13 show the test information curves resulting from the four methods.

### Test Characteristic Curve

Table 2 lists the mean values for the mean square deviation of the five forms from the target test characteristic curve at 61 ability points for all test assembly methods.

**TABLE 2:** *MSD*_TCC_.

	Unimodal (*D* = 0)	Unimodal (*D* = 1)	Bimodal (*D* = 2)	Bimodal (*D* = 3)
MPD	0.227	0.232	0.249	0.268
MID	0.255	0.288	0.368	0.485
MPID	0.193	0.208	0.233	0.247
MIPD	0.197	0.215	0.233	0.263

Regarding the *MSD*_*TCC*_, the MPD method shows its strength of lowering the disparity between target test and assembly tests, resulting in smaller *MSD*_*TCC*_ and outperforming the other methods. The MPID and MIPD methods’ performance is close to that of MPD. Obviously, the MID method has no advantage in TCC.

**FIGURE 14:**
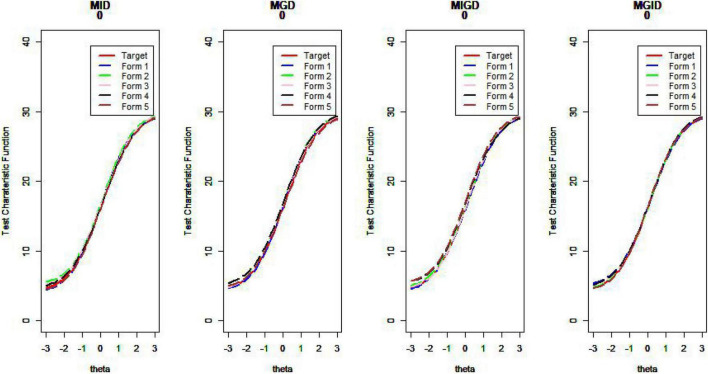
Test characteristic curve (*D* = 0).

**FIGURE 15:**
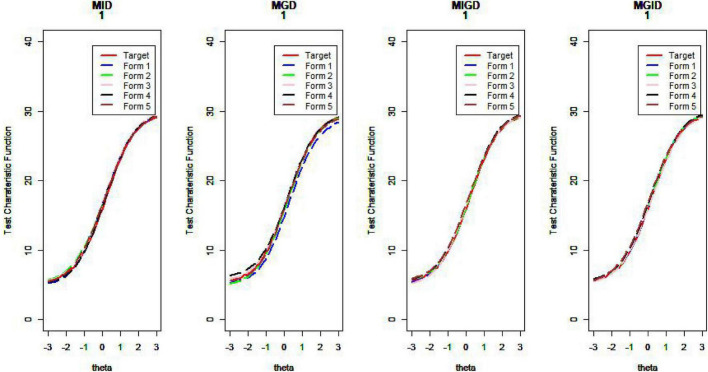
Test characteristic curve (*D* = 1).

**FIGURE 16:**
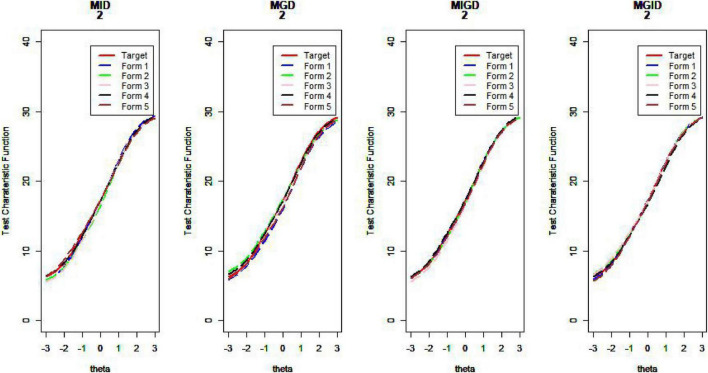
Test characteristic curve (*D* = 2).

**FIGURE 17:**
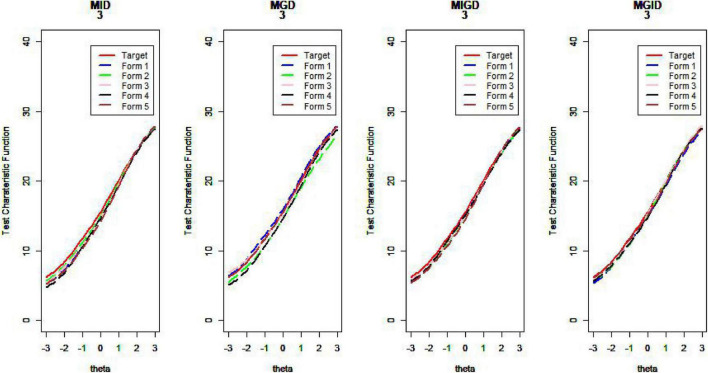
Test characteristic curve (*D* = 3).

Figures 14–17 show the test characteristic curves resulting from the four methods.

## Discussion and Conclusion

As far as the two existing methods are concerned, MPD has advantages in matching TCCs, while MID is superior in matching TICs. Two new methods combining the two methods (the MPID and MIPD method) can not only better match TCCs but also TICs. Although the new method only combines the original method with progressive coefficients, we contend that this research may help test agencies needing to generate multiple test forms for the sake of maintaining test security when administering multiple tests at various locations and times.

There are several reasons for our argument. First, it is undeniable that the two indicators are very important, and they have distinct meanings. The similar TCC results hold for forms that are similar in difficulty; test forms with the same TIF are similar in terms of precision (Ali and Van Rijn, 2016). Hence, we expect that all two indicators will be satisfactory (Chen, 2014). Then, the advantages of the new method are especially reflected in the bimodal distribution conditions. The MID method is susceptible to distribution. Under the bimodal condition, the TCCs matching effect of the two new methods is significantly better than the existing method. Additionally, new methods separating item selection phase into several stages and applying various methods in each stage offer a simple perspective on how to integrate diverse methods’ merits. Finally, this study’s consideration of the different distributions of test information closely matches the reality of test data.

This study has several limitations. The MPID and MIPD methods presented here are simplified versions, and further modification would make them more practical. Other important issues must be addressed in future research, including the setting of λ parameter, to take full advantage of each approach (Liu and Chang, 2018). In addition, it is common knowledge that the ability point specifications can influence the MID method’s results. The issue of whether ability points (–2, –1, 0, 1, 2) suitable for information of unimodal distributions are as appropriate for bimodal distributions deserves additional attention (Chen, 2015). Finally, the item, response, theory (IRT)-based ATA methods proposed in this study focus on information curves of bimodal distributions. Whether the results can be extended to test designs with information curves of other multimodal distributions needs further investigation.

## Data Availability Statement

The original contributions presented in the study are included in the article/Supplementary Material, further inquiries can be directed to the corresponding author.

## Author Contributions

ZH prepared the first draft. LJ, ZP, and KC provided insightful comments that critically improved the quality of the manuscript. All authors contributed to the article and approved the submitted version.

## Conflict of Interest

The authors declare that the research was conducted in the absence of any commercial or financial relationships that could be construed as a potential conflict of interest.

## Publisher’s Note

All claims expressed in this article are solely those of the authors and do not necessarily represent those of their affiliated organizations, or those of the publisher, the editors and the reviewers. Any product that may be evaluated in this article, or claim that may be made by its manufacturer, is not guaranteed or endorsed by the publisher.

## References

[B1] Ali U. S. Van Rijn P. W. (2016). An evaluation of different statistical targets for assembling parallel forms in item response theory. *Appl. Psychol. Meas.* 40 163–179. 10.1177/0146621615613308 29881046 PMC5978482

[B2] Armstrong R. Jones D. Wu I. L. (1992). An automated test development of parallel tests from a seed test. *Psychometrika* 57 271–288. 10.1007/BF02294509

[B3] Baker F. B. Kim S. H. (2017). *The Basics of Item Response Theory Using R.* New York, NY: Springer International Publishing. 10.1007/978-3-319-54205-8

[B4] Brusco M. J. Koehn H. F. Steinley D. (2013). Exact and approximate methods for a one-dimensional minimax bin-packing problem. *Ann. Oper. Res.* 206:611–626. 10.1007/s10479-012-1175-5

[B5] Shao C. Liu S. Yang H. W. Tsai T.-H. (2019). Automated test assembly using SAS operations research software in a medical licensing examination. *Appl. Psychol. Meas.* 44 234–248.32341609

[B6] Chen P. H. (2014). A sampling and classification item selection approach with content balancing. *Behav. Res. Methods* 47 98–106. 10.3758/s13428-014-0452-4 24610145

[B7] Chen P. H. (2015). Three-element item selection procedures for multiple forms assembly:an item machine approach. *Appl. Psychol. Meas.* 40 114–127. 10.1177/0146621615605307 29881042 PMC5982169

[B8] Chen P. H. (2017). Should we stop developing heuristics and only rely on mixed integer programming solvers in automated test assembly? A rejoinder to van der Linden and Li (2016). *Appl. Psychol. Meas.* 41 227–240. 10.1177/0146621617695523 29881090 PMC5978547

[B9] Chen P. H. Chang H. H. Wu H. (2012). Item selection for the development of parallel forms from an IRT-Based seed test using a sampling and classification approach. *Educ. Psychol. Meas.* 72 933–953. 10.1177/0013164412443688

[B10] Chen M. Wang C. R. (2010). Algorithm design based on IRT-guided double boundary test. *J. Minzu Univ. China Nat. Sci. Ed.* 19 53–55. 10.3969/j.issn.1005-8036.2010.03.010

[B11] Cor K. Alves C. Gierl M. (2009). Three applications of automated test assembly within a user-friendly modeling environment. *Pract. Assess. Res. Eval.* 14:23.

[B12] Finkelman M. Kim W. Roussos L. A. (2009). Automated test assembly for cognitive diagnosis models using a genetic algorithm. *J. Educ. Meas.* 46 273–292. 10.1111/j.1745-3984.2009.00081.x

[B13] Finkelman M. Kim W. Roussos L. Verschoor A. (2010). A binary programming approach to automated test assembly for cognitive diagnosis models. *Appl. Psychol. Meas.* 34 310–326. 10.1177/0146621609344846

[B14] Kim S. Y. Lee W. (2020). Classification consistency and accuracy with atypical score distributions. *J. Educ. Meas.* 57 286–310. 10.1111/jedm.12250

[B15] Liu C. J. Chang H. H. (2018). Item selection criteria with practical constraints in cognitive diagnostic computerized adaptive testing. *Edu. Psychol. Meas.* 79 335–357. 10.1177/0013164418790634 30911196 PMC6425095

[B16] Ramai D. Ofosu A. Lai J. K. Gao Z. H. Adler D. G. (2021). Fibrolamellar hepatocellular carcinoma: a population-based observational study. *Dig. Dis. Sci.* 66 308–314. 32052215 10.1007/s10620-020-06135-3

[B17] Revuelta J. Ponsoda V. (1998). A comparison of item exposure control methods in computerized adaptive testing. *J. Educ. Meas.* 35 311–327.

[B18] Steinley D. McDonald R. P. (2007). Examining factor score distributions to determine the nature of latent spaces. *Multivar. Behav. Res.* 42 133–156. 10.1080/00273170701341217 26821079

[B19] Tang H. (2018). Research on the causes of the bimodal distribution of students’ academic achievement based on QMAS. *Comput. Fan* 109:232.

[B20] van der Linden W. (2005). *Linear Models for Optimal Test Design.* New York, N Y: Springer, 408. 10.1198/jasa.2006.s148

[B21] Wang S. Yi Z. Zheng C. Su Y. H. Li P. (2016). An automated test assembly design for a large-scale chinese proficiency test. *Appl. Psychol. Meas.* 40 233–237. 10.1177/0146621616628503 29881050 PMC5978481

[B22] Wang W. Y. Xiong J. Song L. H. Zheng J. J. Hu H. Y. (2021). MPI method of double-question matching and its application in parallel test paper generation. *J. Jiangxi Norm. Univ. Nat. Sci. Ed.* 45 118–125. 10.16357/j.cnki.issn1000-5862.2021.02.02

[B23] Xiong J. H. Ding S. L. Qi S. Q. Dai H. Q. (2002). Use test information to analyze test paper quality. *J. Jiangxi Norm. Univ.* 26, 225–228.

[B24] Xu X. Guo M. Z. Shi F. L. (2013). Simulation of bimodal data distribution. *J. Yunan Norm. Univ.* 33, 46–51.

