# Supplementary material for: Two Test Assembly Methods With Two Statistical Targets
Source: Front Psychol. 2022 Feb 11;13:786772. doi: 10.3389/fpsyg.2022.786772 (PMC8873185; doi:10.3389/fpsyg.2022.786772)
Supplement: Supplementary file 5 [file Data_Sheet_5.docx]

**Appendix**

The first three tables show the results (average of 1,000 iterations) using a bimodal mixing ratio of 5:5. The figures in the previous section are taken from one of the 1,000 sets of results.

Table 1. *MSD*_TIC_

|  | Unimodal (*D* = 0) | Uimodal (*D* = 1) | Bimodal (*D* = 2) | Bimodal (*D* = 3) |
| --- | --- | --- | --- | --- |
| MPD | 0.395 | 0.393 | 0.429 | 0.424 |
| MID | 0.316 | 0.324 | 0.361 | 0.397 |
| MPID | 0.303 | 0.306 | 0.336 | 0.343 |
| MIPD | 0.298 | 0.300 | 0.332 | 0.347 |


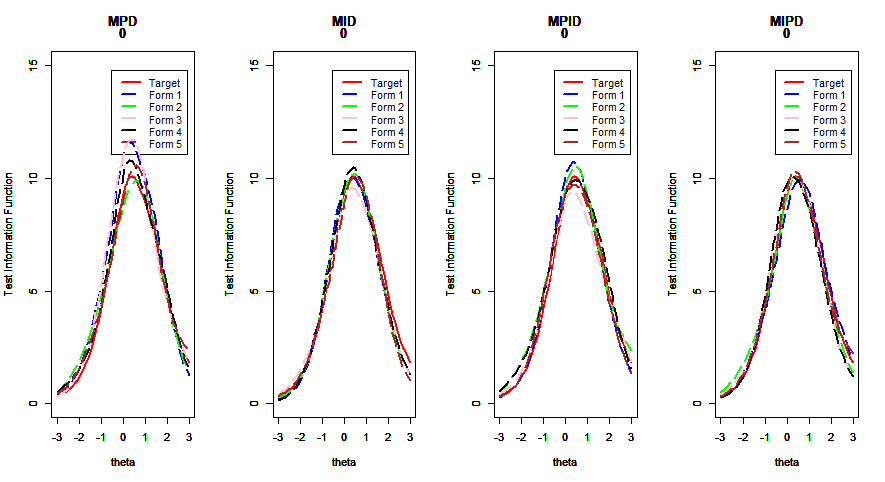


Figure 1. Test information curve (*D* = 0)


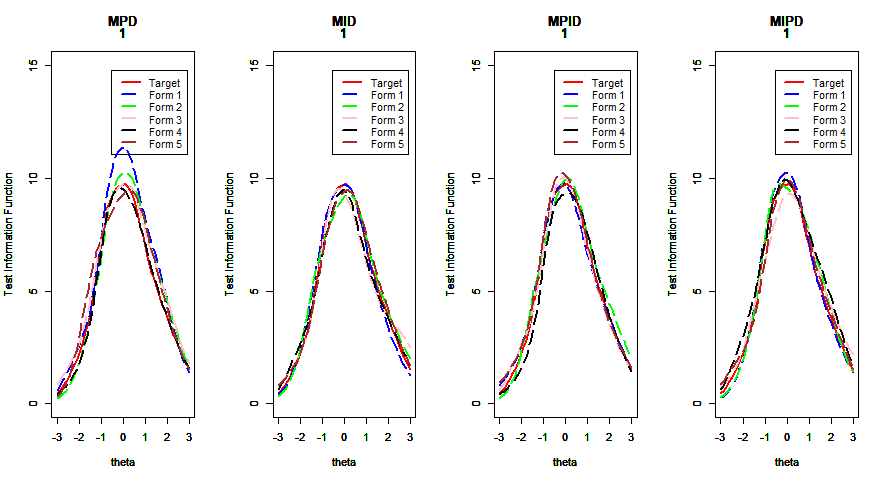


Figure 2. Test information curve (*D* = 1)


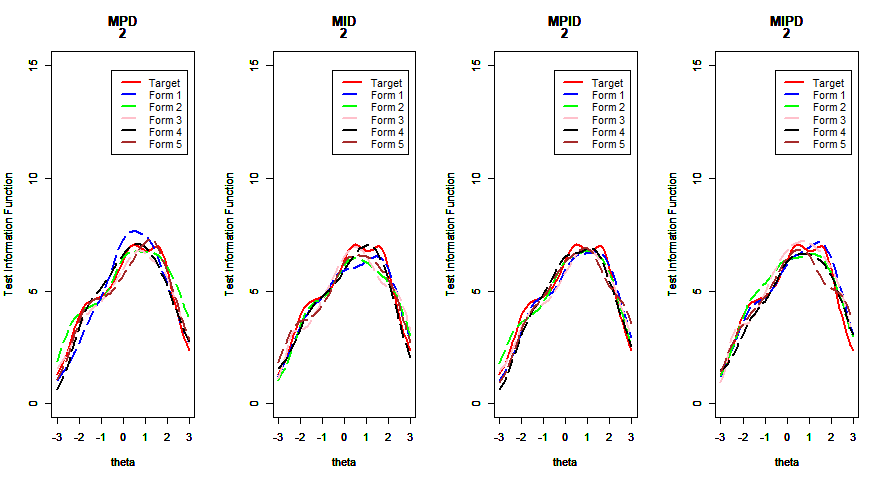


Figure 3. Test information curve (*D* = 2)


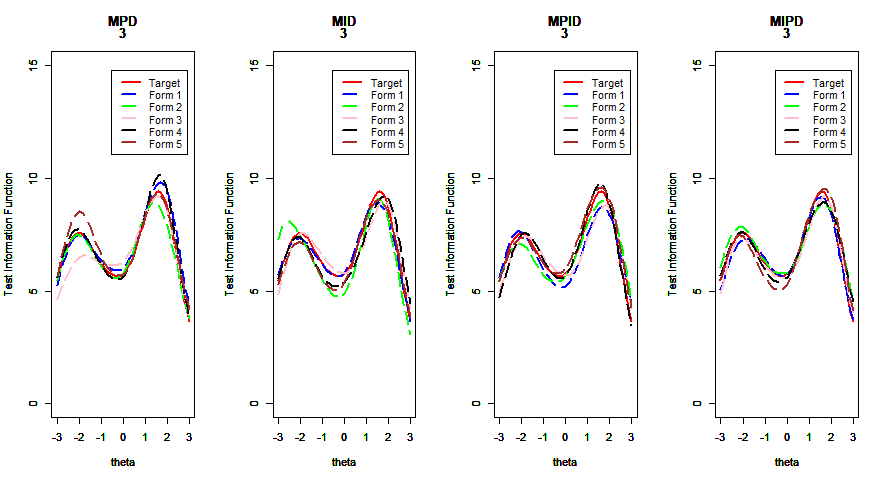


Figure 4. Test information curve (*D* = 3)

Table 2. *MSD*_TCC_

|  | Unimodal (*D* = 0) | Uimodal (*D* = 1) | Bimodal (*D* = 2) | Bimodal (*D* = 3) |
| --- | --- | --- | --- | --- |
| MPD | 0.215 | 0.223 | 0.232 | 0.231 |
| MID | 0.254 | 0.275 | 0.345 | 0.457 |
| MPID | 0.193 | 0.203 | 0.214 | 0.222 |
| MIPD | 0.195 | 0.204 | 0.223 | 0.239 |


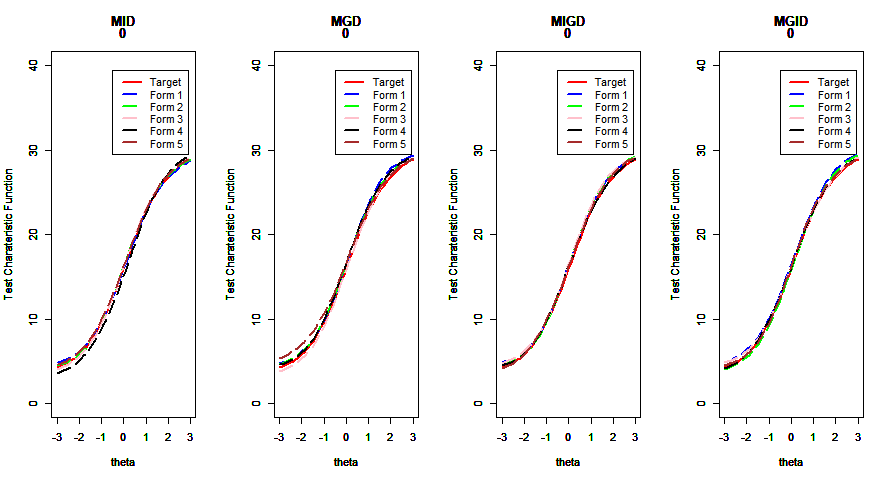


Figure 5. Test characteristic curve (*D* = 0)


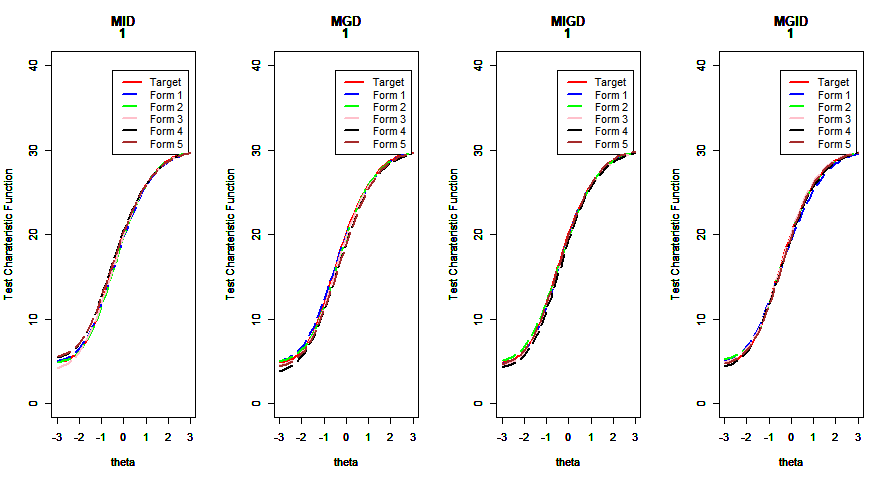


Figure 6. Test characteristic curve (*D* = 1)


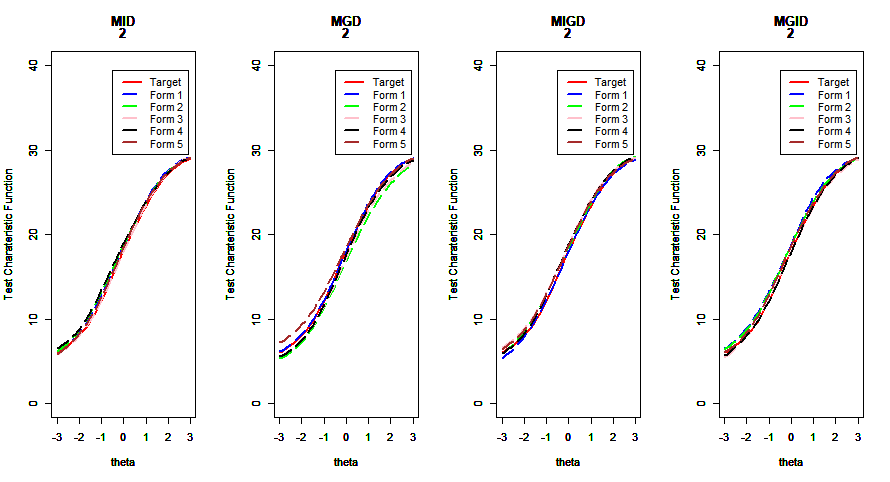


Figure 7. Test characteristic curve (*D* = 2)


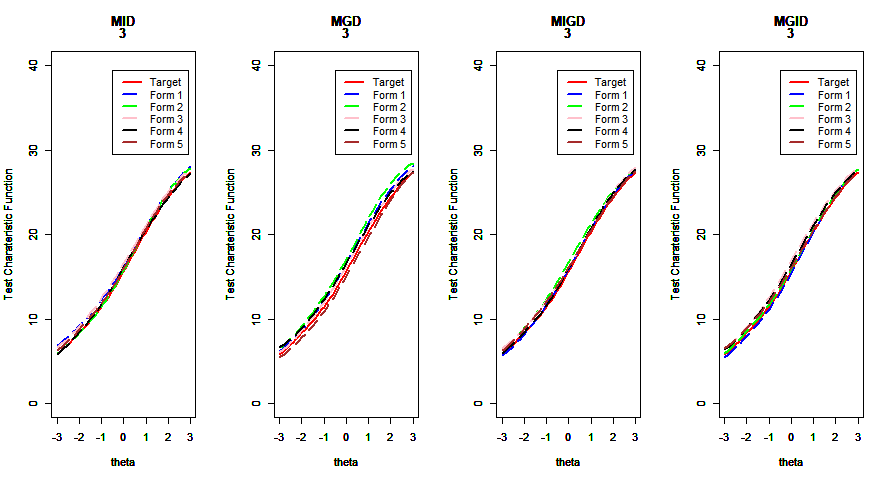


Figure 8. Test characteristic curve (*D* = 3)

The next three tables show the results (average of 1,000 iterations) using a bimodal mixing ratio of 7:3. The figures in the previous section are taken from one of the 1,000 sets of results.

Table 4. *MSD*_TIC_

|  | Unimodal (*D* = 0) | Uimodal (*D* = 1) | Bimodal (*D* = 2) | Bimodal (*D* = 3) |
| --- | --- | --- | --- | --- |
| MPD | 0.402 | 0.419 | 0.421 | 0.429 |
| MID | 0.315 | 0.335 | 0.353 | 0.389 |
| MPID | 0.301 | 0.322 | 0.331 | 0.352 |
| MIPD | 0.302 | 0.318 | 0.323 | 0.344 |


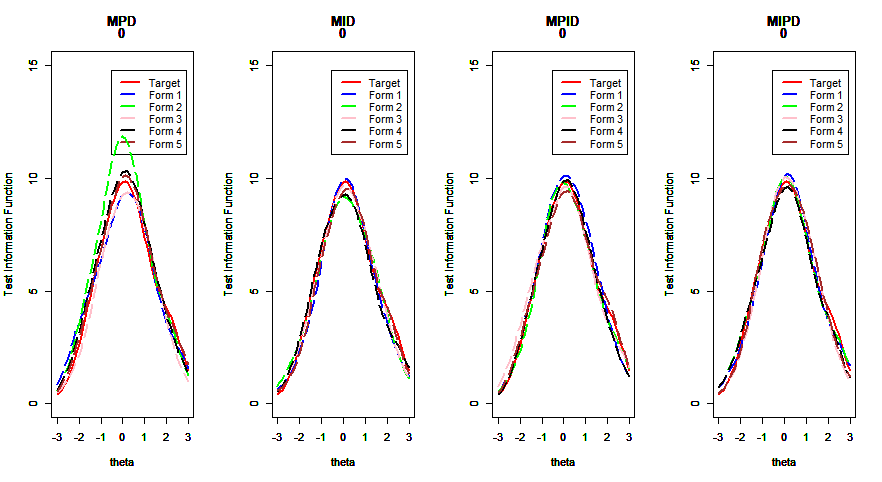


Figure 9. Test information curve (*D* = 0)


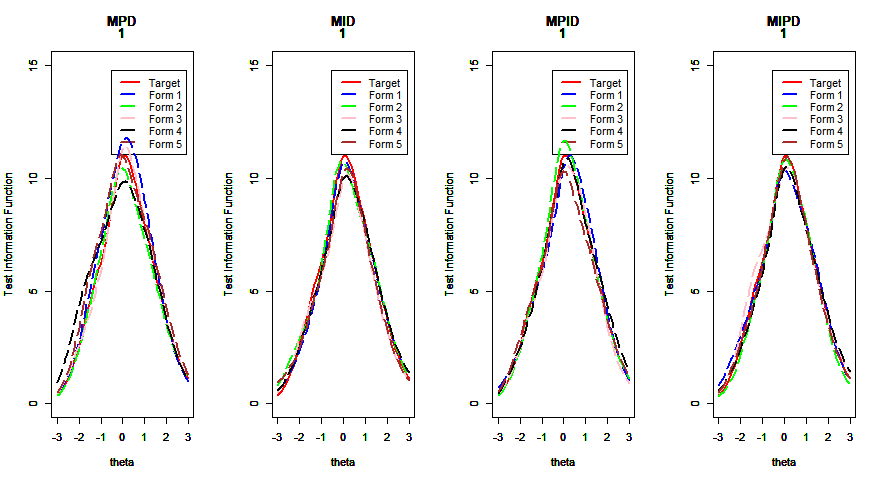


Figure 10. Test information curve (*D* = 1)


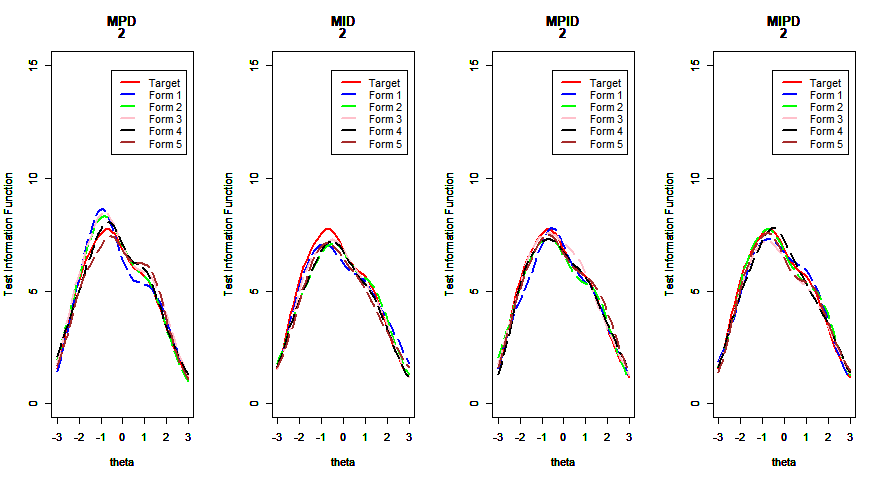


Figure 11. Test information curve (*D* = 2)


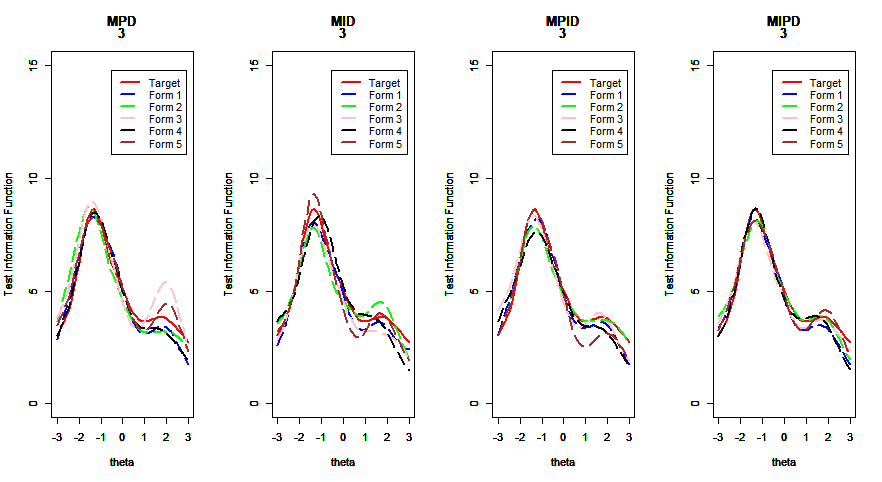


Figure 12. Test information curve (*D* = 3)

Table 5. *MSD*_TCC_

|  | Unimodal (*D* = 0) | Uimodal (*D* = 1) | Bimodal (*D* = 2) | Bimodal (*D* = 3) |
| --- | --- | --- | --- | --- |
| MPD | 0.217 | 0.213 | 0.213 | 0.207 |
| MID | 0.260 | 0.271 | 0.324 | 0.412 |
| MPID | 0.195 | 0.194 | 0.200 | 0.201 |
| MIPD | 0.201 | 0.198 | 0.208 | 0.214 |


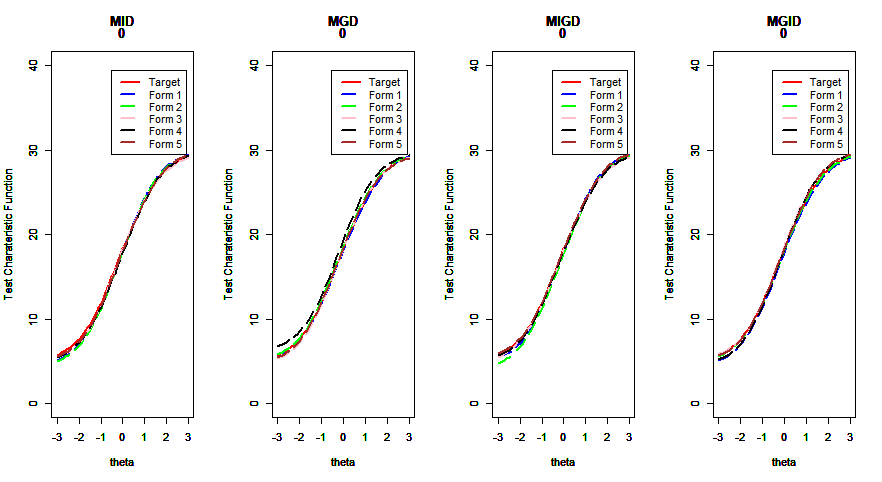


Figure 13. Test characteristic curve (*D* = 0)


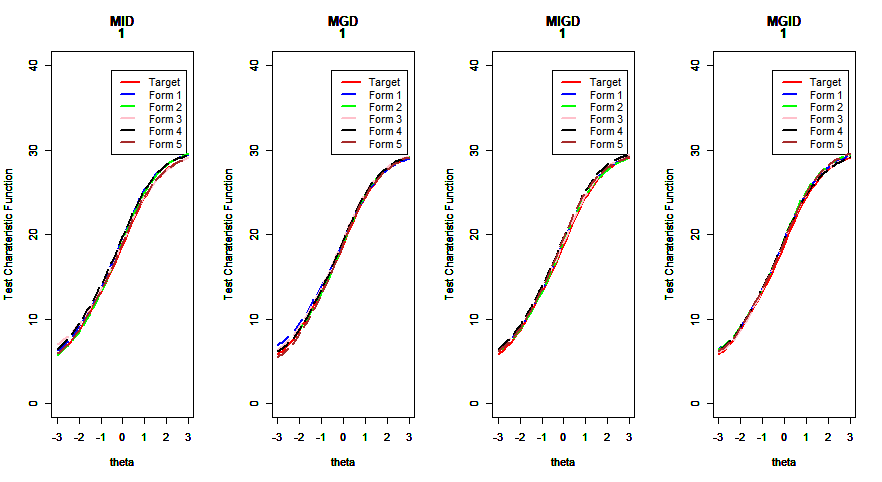


Figure 14. Test characteristic curve (*D* = 1)


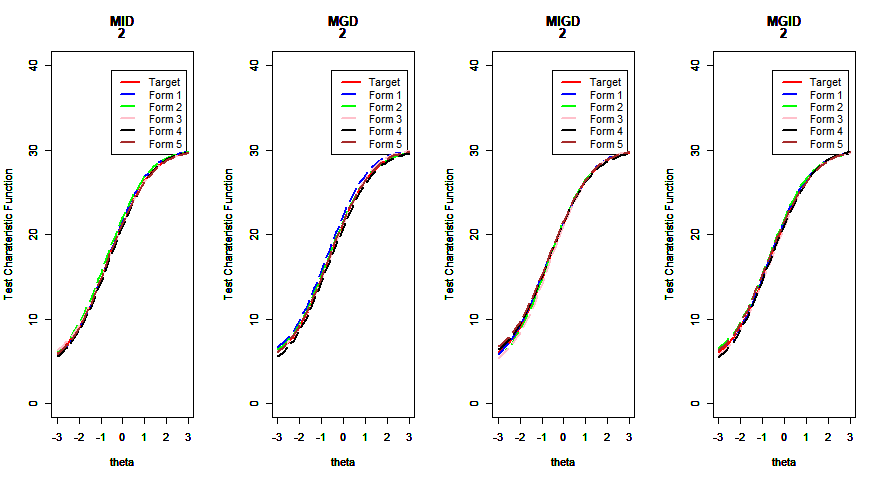


Figure 15. Test characteristic curve (*D* = 2)


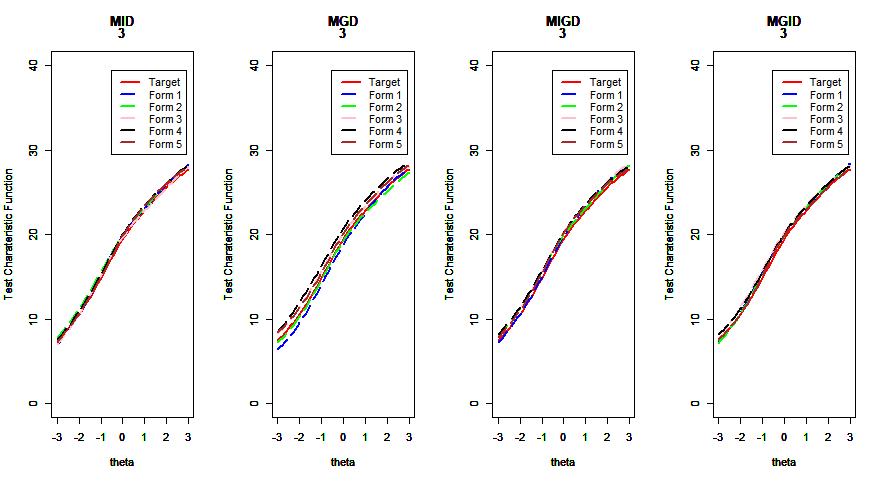


Figure 16. Test characteristic curve (*D* = 3)
